# Glutathione and copper ions as critical factors of green plant regeneration efficiency of triticale *in vitro* anther culture

**DOI:** 10.3389/fpls.2022.926305

**Published:** 2022-07-28

**Authors:** Piotr T. Bednarek, Renata Orłowska, Dariusz R. Mańkowski, Janusz Zimny, Krzysztof Kowalczyk, Michał Nowak, Jacek Zebrowski

**Affiliations:** ^1^Plant Breeding and Acclimatization Institute-National Research Institute, Radzików, Poland; ^2^Institute of Plant Genetics, Breeding and Biotechnology, University of Life Sciences in Lublin, Lublin, Poland; ^3^Institute of Biology and Biotechnology, University of Rzeszow, Rzeszow, Poland

**Keywords:** androgenesis, copper, glutathione, regeneration efficiency, triticale

## Abstract

Plant tissue culture techniques are handy tools for obtaining unique plant materials that are difficult to propagate or important for agriculture. Homozygous materials derived through *in vitro* cultures are invaluable and significantly accelerate the evaluation of new varieties, e.g., cereals. The induction of somatic embryogenesis/androgenesis and the regeneration and its efficiency can be influenced by the external conditions of tissue culture, such as the ingredients present in the induction or regeneration media. We have developed an approach based on biological system, molecular markers, Fourier Transform Infrared spectroscopy, and structural equation modeling technique to establish links between changes in sequence and DNA methylation at specific symmetric (CG, CHG) and asymmetric (CHH) sequences, glutathione, and green plant regeneration efficiency in the presence of variable supplementation of induction medium with copper ions. The methylation-sensitive Amplified Fragment Length Polymorphism was used to assess tissue culture-induced variation, Fourier Transform Infrared spectroscopy to describe the glutathione spectrum, and a structural equation model to develop the relationship between sequence variation*, de novo* DNA methylation within asymmetric sequence contexts, and copper ions in the induction medium, as well as, glutathione, and green plant efficiency. An essential aspect of the study is demonstrating the contribution of glutathione to green plant regeneration efficiency and indicating the critical role of copper ions in influencing tissue culture-induced variation, glutathione, and obtaining green regenerants. The model presented here also has practical implications, showing that manipulating the concentration of copper ions in the induction medium may influence cell function and increases green plant regeneration efficiency.

## Introduction

The regeneration of plants by tissue culture has long been considered a way to obtain material identical to the donor plant (Metakovsky et al., 1987). This way of plant obtention is associated with the presence of a well-known phenomenon called somaclonal variation (Larkin and Scowcroft, 1981; Scowcroft, 1985) or tissue culture-induced variation (TCIV). The early studies carried out in the 80s and 90s of the 20^th^ century demonstrated its putative genetic background (Evans et al., 1984). Some authors interrogated the phenomenon, suggesting that the variation is due to pre-existing variation in explants (Skirvin et al., 1994). Strictly defined biological materials encompassing doubled haploid donor plants that underwent at least a single generative cycle and regenerants derived *via* embryo or androgenesis are required (Orłowska and Bednarek, 2020) to limit the variation and identify variants that could be assigned exclusively to the tissue culture-induced variation (Orłowska and Bednarek, 2020). The other limitation of studies on TCIV was the lack of molecular systems capable of identifying sequence and DNA methylation changes during a single experiment and quantifying different changes. The development of the methylation-sensitive Amplified Fragment Length Polymorphism (metAFLP) approach (Bednarek et al., 2007) overcame the restriction. If molecular data are treated quantitatively, many next-generation sequencing (NGS) technologies might be used for such studies. Furthermore, NGS-based markers may deliver additional information on putative genes involved in TCIV. For example, the Diversity Arrays Technology Methylation Analysis (DArTseqMet; Pereira et al., 2020) or Methyl-seq (Brunner et al., 2009) could be indicated among the methods. However, the NGS-based methods are relatively expensive, and thus, their application is not always justified.

Not long ago, the TCIV was linked to DNA sequence changes and DNA methylation alterations (Machczyńska et al., 2014). The latter is due to the cell reprogramming (Jullien and Berger, 2010) involving DNA demethylation and *de novo* methylation. Cytosine in plants may be methylated in several contexts, including symmetric (CG, CHG) and asymmetric (CHH) sequences (with H any nucleotide but G). DNA methylation pattern is reestablished either by DNA replication or/and epigenetic (RNA-directed) mechanisms (Finnegan et al., 1993; Lindroth et al., 2001; Chen and Li, 2004; Goll and Bestor, 2005; Cuerda-Gil and Slotkin, 2016). RNA-directed DNA methylation (RdDM) is the only pathway that controls *de novo* DNA methylation in all sequence contexts in plants (Matzke and Mosher, 2014) and maintains DNA methylation patterns. However, different sequence contexts have their own pathways, i.e., methyltransferase MET1 methylates CG context, chromomethylase 3 (CMT3), and CMT2 participate in cytosine methylation within CHG and CHH contexts, respectively (Stroud et al., 2014; Bartels et al., 2018; Zhang et al., 2018). Furthermore, the RdDM pathway is exclusively responsible for the methylation of previously unmethylated cytosines. Under anther tissue culture conditions, the RdDM epigenetic pathway involving CHH sequence methylation gives the only opportunity for methylation of such sequences. In contrast, the other contexts could be reestablished during DNA replication. Recent studies have revealed that sequence variation may exceed DNA methylation change (Orłowska et al., 2022) or *vice versa* (Orłowska et al., 2021). It was also shown that there are differences in sequence and methylation patterns within symmetric and asymmetric contexts, possibly indicating the role of methylation in green plant regeneration efficiency (GPRE) (Bednarek et al., 2021a). However, studies of TCIV based on sequence and DNA methylation changes indicated the phenomenon’s complexity but could not explain its nature (Bednarek and Orłowska, 2020b; Ghosh et al., 2021; Bednarek et al., 2021b). At least to some degree, such the opportunity may be born using Fourier Transform Infrared (FTIR) spectroscopy, which provides signals of relevant functional groups and thus allows the identification of chemical compounds in a sample. A large number of FTIR applications have been reported for studies with plant tissues, mainly focused on the effect of various growth conditions or abiotic stresses on biochemical phenotype (Lahlali et al., 2014; Butler et al., 2015; Pérez-Rodríguez et al., 2016; Sharma and Uttam, 2016; Rana et al., 2018; da Silva Leite et al., 2018; Liu et al., 2019). The method was also used in studies on barley anther *in vitro* tissue cultures (Bednarek et al., 2021a).

Preliminary studies of TCIV showed its linkage with GPRE (Bednarek et al., 2021a). Moreover, the two phenomena’ origin might be based on biochemical processes (Maraschin et al., 2002; Kumari et al., 2009; Makowska and Oleszczuk, 2014). It was suggested that the callose interstitial layer in microspores might be necessary for new plant regeneration efficiency (Rivas-Sendra et al., 2019; Dubas et al., 2021; Zieliński et al., 2021). The callose layer under starvation conditions and in darkness (typical conditions for plant regeneration *via* androgenesis) may be the only source of glucose pumping glycolysis and allowing the cell’s surveillance (Roulin et al., 2002). Such data were presented for barley regenerants, where the composition of the media (strictly induction media) was manipulated at the stage of androgenesis induction (Bednarek et al., 2020). It cannot be excluded that callose may also have an impact on microspore embryogenesis (Parra-Vega et al., 2015) possibly having impact on GPRE. It was also demonstrated that S-adenosyl-L-methionine (SAM), the product of the Yang cycle, is also vital in the process as it is responsible for *de novo* DNA methylation at least in barley (Bednarek et al., 2020). The GPRE may also depend, i.e., on copper/zinc superoxide dismutase (Cu/Zn SOD), glutathione (GSH), ascorbic acid, melatonin, and reactive oxygen (ROS) or reactive nitrogen species (RNS) scavengers, as they may minimize a negative effect of radicals or abiotic stress in general (Rajput et al., 2021). Gaining competence, induction, and development of somatic embryos is associated with a gradual increase in SOD activity observed in peanut (*Arachis hypogaea*) explants (Qiusheng et al., 2005). In addition, SOD can also affect cell proliferation during somatic embryogenesis and shoot organogenesis from cultured leaf segments of gladiolus (*Gladiolus hybridus* Hort; Gupta and Datta, 2003). Therefore, it can be speculated that Cu/Zn SOD may affect GPRE. Similarly, positive effects of ROS (RNS) scavengers, i.e., glutathione, ascorbic acid, and melatonin, can be expected to improve plant derivation through *in vitro* cultures. An example is an addition of glutathione to the induction medium which resulted in increased microspore embryogenesis in triticale and wheat and increased green plant numbers in recalcitrant genotypes (Asif et al., 2013). Also, medium supplementation with ascorbic acid in rapeseed (*Brassica napus* L.) and sweet pepper (*Capsicum annuum* L.) cultures improved microspore embryogenesis (Hoseini et al., 2014; Heidari-Zefreh et al., 2019). In contrast, an increase in endogenous melatonin concentration correlated with an increase in *de novo* root formation in explants of St. John’s Wort (*Hypericum perforatum* L.) (Murch et al., 2001). GSH is considered an important antioxidant (Szalai et al., 2009). The molecule binds copper Cu(I) *in vivo* (Freedman et al., 1989; Maryon et al., 2013) and Cu(II)/Cu(I) pair takes part in the scavenging of ROS (Tamasi et al., 2018). Moreover, GSH is also a critical epigenetic factor affecting enzymatic activity for DNA methylation, a posttranscriptional modifier of histone code, miRNAs expression (García-Giménez et al., 2017), and is a component of the glutathione-ascorbate cycle responsible for the cell redox (Pandey et al., 2015). Furthermore, glutathione may impact embryogenesis and green plant regeneration, as was shown for rye (Żur et al., 2019) and triticale (Zieliński et al., 2020). However, the mechanism *via* which GSH influences GPRE is not apparent.

Most biochemical pathways include metal ion cofactors, i.e., manganese (Burnell, 1988), copper (Cohu and Pilon, 2010), or zinc ions (Castillo-González et al., 2018). Copper significance in the TCIV was recently demonstrated in barley anther (Bednarek et al., 2021a) and zygotic embryo (Orłowska et al., 2021) and triticale anther cultures (Orłowska et al., 2022). Copper ions can affect the efficiency of green plant regeneration by improving the survival rate of microspores and affecting the synchronization of the first division of microspores (Wojnarowiez et al., 2002). In addition, increasing the concentration of copper ions in tissue culture media positively affects somatic embryogenesis (Dordević et al., 2019) or androgenesis (Makowska et al., 2017; Bednarek and Orłowska, 2020c). Supplementing the media used in plant tissue cultures with Cu(II) can improve the green to albino regenerants (Nuutila et al., 2000; Brew-Appiah et al., 2013), and lowering Cu(II) levels in induction media (IM) was associated with a decrease in DNA methylation in barley regenerants. On the other hand, an increase in Cu(II) levels led to an increase in the frequency of DNA sequence changes in barley regenerants (Bednarek and Orłowska, 2020a). Although the effect of copper ions on the regeneration efficiency of green plants is known and described, the biochemical basis for this phenomenon is not fully elucidated. Furthermore, manipulating the ion concentrations in the IM may affect biochemical pathways (Bednarek et al., 2020), TCIV (Orłowska, 2021), and GPRE (Orłowska et al., 2020). However, the complexity of the phenomena requires the implementation of sophisticated statistical methods allowing linking varying aspects of the phenomenon in theoretical models. Machine learning is one of the alternatives (Sadat-Hosseini et al., 2022). Its main advantage is that no theoretical model is needed for analysis. However, it does not allow for the verification of theoretical models, limiting the understanding of the nature of the phenomenon. Mediation analysis (Orłowska et al., 2021), relying on regression techniques, may be a solution when searching for simple relationships. Structural equation modeling (SEM) involving probabilistic approaches could be used when complex relationships are considered. Both mediation and SEM methods are phenomenon-based analyses. They proved to be supportive in barley anther (Bednarek and Orłowska, 2020a) and zygotic embryo culture (Orłowska et al., 2021) and triticale anther tissue cultures (Orłowska et al., 2022).

Assuming that: (1) a specially designed biological system limiting pre-existing variation is used; (2) molecular data based on the metAFLP technique may deliver quantitative characteristics concerning sequence and DNA methylation changes affecting both symmetric and asymmetric sequence contexts; (3) information on biochemical pathway compounds is available analyzing FTIR spectra; (4) copper ions act as cofactors of enzymatic reactions affecting biochemical pathways; and (5) GSH is the cellular antioxidant that may impact on epigenetic mechanisms; we suspected that all the characteristics mentioned above might be linked to GPRE in the form of a simple theoretical model of *in vitro* anther culture plant regeneration.

## Materials and methods

### Plant material

**Figure 1:**
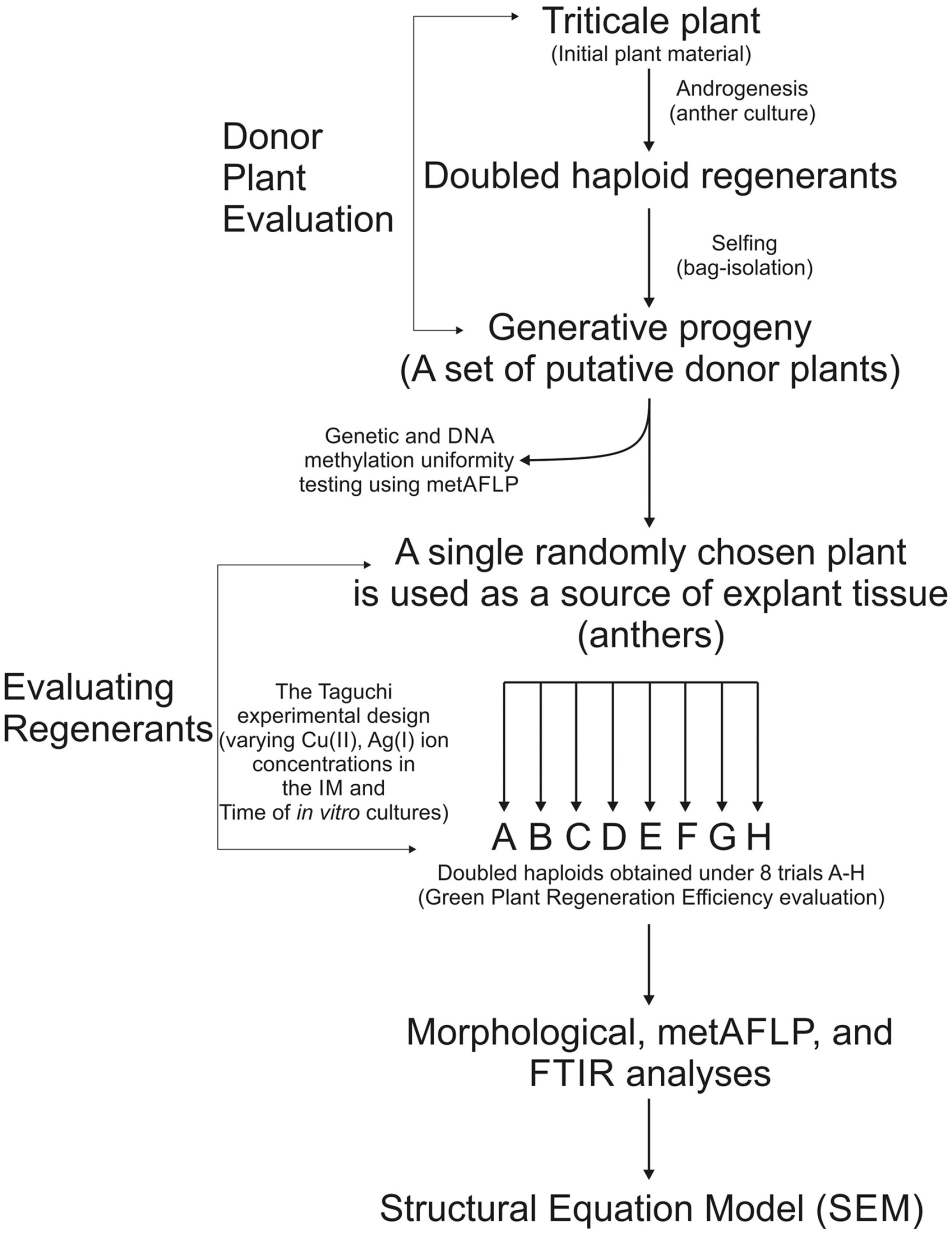
Plant material evaluation and analysis. A randomly chosen plant of triticale was used as a source of tissue explants (anthers) to obtain doubled haploid (DH) plants that were self-isolated (bagged) to obtain generative progeny (a set of putative donor plants). The progeny was analyzed via the metAFLP approach for the sequence variation and DNA methylation pattern uniformity. A single randomly chosen progeny (donor plant) was used as a source of anthers to obtain regenerants under the varying concentration of Cu(II), Ag(I) present in the IM, and different Time of in vitro anther cultures according to the Taguchi experimental design. In total, eight such experiments were conducted where the A one reflected control conditions and the B-H required by the approach. The regenerants for each trial were counted, and GPRE was evaluated. Visual morphological inspection, metAFLP, and Fourier Transform Infrared (FTIR) analyses were performed for each regenerant, and the evaluated data was implemented for structural equation modeling.

Winter hexaploid triticale (X *Triticosecale* spp. Wittmack ex *A. Camus* 1927) genotype T28/2 derived from cv. Presto × cv. Mungis cross was used in this study. The detailed procedure of preparing donor plants through *in vitro* cultures and the generative cycle has been described previously (Pachota et al., 2022). First, seeds of initial triticale plants (Figure 1), available by dr. Sylwia Oleszczuk (Plant Breeding and Acclimatization Institute-National Research Institute, Radzików, Poland), were sown and grown under controlled conditions until the microspores in the spikes were mid- to the uni-nucleate stage. Tillers were then sheared and subjected to cold stress for 21 days at 4°C and darkness. After this time, anthers were plated onto induction medium (IM) 190–2 (Zhuang and Xu, 1983) with 90 g l^−1^ maltose and 438 mg l^−1^ glutamine supplemented with 2 mg l^−1^ 2,4-dichlorophenoxyacetic acid and 0.5 mg l^−1^ kinetin and incubated in the dark at 26°C. When the first callus and embryo-like structures appeared, they were transferred onto a regeneration medium 190–2 (Zhuang and Xu, 1983) supplemented with 0.5 mg l^−1^ naphthalene acetic acid and 1.5 mg l^−1^ kinetin and incubated under photoperiod conditions (16 h/8 h light/dark) at 26°C. Next, green regenerants were transferred to glass flasks with an N6I rooting medium (Chu, 1978) supplemented with 2 mg l^−1^ indole-3-acetic acid. Next, plantlets were planted in pots and grown in greenhouse conditions. The chromosome doubling occurred spontaneously. The diploidization was estimated, considering regenerants morphology (plant growth; leaf shape, color, and width; tillering mode; and spike number) and comparing it with the initial plants. Finally, spikes were self-pollinated in a pool of randomly selected regenerants, and plants were grown to maturity. The seeds of doubled haploid (DH) regenerants were sown and grown, and those plants—generative progenies derived from DH regenerants were used as donor plants to obtain regenerants in different *in vitro* conditions to test the impact of various concentrations of CuSO_4_ × 5H_2_O and AgNO_3_ in the IM and the time of incubation explants on them. Modifications of *in vitro* culture conditions concerned the induction process of androgenesis; hence, only the IMs were supplemented with different concentrations of CuSO_4_ × 5H_2_O and AgNO_3_, and different incubation times of anthers on the IM were used. However, the experiment’s regeneration media and incubation time of callus, embryogenic structures, and somatic embryos were identical.

Twenty-four donor plants were the source of explants for anther cultures (Figure 1). The obtention of regenerants in the presence of different concentrations of copper Cu(II) and silver Ag(I) ions in the IM and the time of anther incubation on IM was tested. The same induction (190–2) and regeneration (190–2) media were used as in the case of obtaining the donor plants; however, the IMs were modified (Figure 1). As salts, copper and silver ions were added to IM: CuSO_4_ × 5H_2_O at 0.1, 5, 10 μM, and AgNO_3_ at 0, 10, and 60 μM concentrations. The incubation time was 35, 42, and 49 days, covering the time from plating anthers on the IM to calli, embryo-like structures, or somatic embryo collection and transferring them on regeneration media. Eight different A-H trial conditions were prepared to test the effect of Cu(II) and Ag(I) ions, and time on the tissue culture-induced variation and the number of green regenerants obtained. In each trial, the number of green regenerants obtained per 100 plated anthers was estimated and referred to as green plant regeneration efficiency (GPRE). The doubling of the number of chromosomes in regenerants occurred spontaneously. Therefore, the assessment of diploidization was estimated visually by examining plant height; leaf shape, color, and width; tillering mode; spike number; and seed setting ability and comparing it with the donor plants. Finally, regenerants that were the doubled haploid plants representing all trials (A-H) from a single donor plant were selected for analysis.

### DNA isolation and metAFLP approach

According to the manufacturer’s instructions, a standard DNA isolation kit (Qiagen, Hilding, Germany) was used to extract DNA from leaf samples of donor plants and regenerants. For metAFLP, two samples from one plant at 500 ng of genomic DNA were prepared. The entire metAFLP procedure was performed using Acc65I and MseI and KpnI and MseI enzyme pairs (forming Acc65I/MseI and KpnI/MseI platforms) to cut genomic DNA (Machczyńska et al., 2014). The restriction enzymes used in the experiment presented here recognize the same nucleotide sequence in DNA but exhibit different sensitivity to the presence of methylated cytosine. The KpnI enzyme is not sensitive to methylated cytosine in the restriction site. In contrast, the Acc65I enzyme is sensitive to a methyl group in the cytosine at the restriction site and adjacent sequences and does not digest DNA when the cytosine is methylated. The DNA band profiles generated in the Acc65I/MseI and KpnI/MseI platforms are used for marker counting. When there is cytosine methylation at the restriction site, the pattern of AFLP band profiles generated by the two restriction enzymes will differ for the same DNA (Supplementary Figure S1). The molecular profiles amplified on the donor (D) plant and its regenerants (R) DNAs for both sets of restriction enzymes were transformed into a zero–one matrix, where the presence of a band was encoded as “1.” In contrast, the absence was encoded as “0.” Thus, the band profile for donor and regenerant can be encoded as a 4-digit code, where the first two digits reflect the presence or absence of a DNA fragment in the Acc65I/MseI for D and R, and the second two reflect the markers for D and R in the KpnI/MseI. There are 16 possible combinations of DNA patterns for D and R and two sets of restriction enzymes from 0000 to 1111. Each code is the result of a specific genetic background of the sequence (SV), demethylation (DMV), and *de novo* methylation (DNMV) events, as well as, for example, sites unaffected by any change between donor and regenerant. Codes reflecting the same type of event collectively are used to provide quantitative features of the metAFLP approach. The binary codes (event types) can be converted into variations after dividing them by the sum of all detected events and expressed in percentages. Finally, the juxtaposition of band profiles from the donor and regenerant plant allows quantification of individual metAFLP traits (SV, DNM, and DNMV) and tissue culture-induced variation (TCIV). A detailed description of the quantitative characterization of TCIV, including formulas for estimating SV, DMV, DNMV, and TCIV, was described in detail earlier (Bednarek et al., 2007; Machczyńska et al., 2014), with some modifications accompanied by an Excel file capable of performing the respective calculations (Orłowska and Bednarek, 2020).

Utilizing specially designed selective primers targeting symmetric and asymmetric DNA sequences assessed by the metAFLP technique allows the identification of contexts of CG, CHG, and CHH (where H is any base except G) methylation changes. The distinction is possible because Acc65I is sensitive to the DNA methylation of its restriction site and nearest vicinity. Thus, selective primers complementing different surroundings could be designed. For example, selective primers ending with any combination of two A and T at their 3′ ends should amplify the asymmetric CHH sequence. Furthermore, the metAFLP primers ending with the-CHG and those with the-CG sequence reflect a symmetric context. Thus, all types of changes affecting sequence contexts could be evaluated applying the metAFLP approach (Orłowska and Bednarek, 2020).

### Attenuated total reflectance (ATR)–Fourier transform infrared (FTIR) spectroscopy

Samples for the mid-infrared spectroscopy were lyophilized (laboratory freeze dryer *Alpha 1–4* LSC Christ; Polygen, Østerode, Germany) and homogenized into powder (ball mill, MM 400, Retsch, Haan, Germany). The measurements were performed using the iZ10 module of the Nicolet iN10 MX infrared imaging microscope (Thermo Fisher Scientific, Waltham, MA, United States), equipped with a deuterated triglycine sulfate (DTGS) detector and a KBr beam splitter. Sixty-four spectra per sample were collected in the Attenuated Total Reflectance (ATR) mode at 4 cm^−1^ resolution in the wavenumber range between 600 and 4,000 cm^−1^ using the one-bounce diamond crystal and the ATR accessory (Smart Orbit, Thermo Scientific, Madison, WI, United States). The surface of the diamond crystal was cleaned with water or propanol before each measurement to remove residuals from previous samples. The spectra were recorded, averaged, and baseline corrected using OMNIC software (v.9.0, Thermo Fischer Scientific Inc.). The normalization of the unit area within the 1,800–900 cm^−1^ wavenumber region, statistics (mean, SD), and plots of spectra were performed with ChemoSpec (Hanson, 2017) package in the R programming language (R Core Team, 2021). Apart from seedling leaves, standards of solid L-glutathione in oxidized (GSSG) and reduced (GSH) states (purchased from Sigma Aldrich (#G4376 and #G4251, respectively)) were analyzed to get a spectral reference of the compound.

### Statistics

The descriptive statistics and Pearson’s correlations were evaluated in SPSS v.28 (IBMCorp, 2021). In addition, structural equation modeling (SEM), including model characteristics, was conducted in SPSS v.28 using AMOS v.27 (Arbuckle, 2014).

## Results

The generative progeny (24 plants) of selfed doubled haploid triticale regenerant obtained in anther cultures from microspores was uniform in morphological traits (height, leaf size, tillering, and seed set) assessed visually. All donor plants were used to evaluate regenerants in eight tested trials (A–H). However, under *in vitro* culture conditions [differing in the concentration of Cu(II), Ag(I) ions in IM] and the time of *in vitro* anther cultures, only a single donor plant allowed regeneration of the highest number of regenerants (37 individuals) in all trials simultaneously. These regenerants were used for analysis employing metAFLP and ATR–FTIR techniques. All regenerants derived *via* androgenesis did not differ in morphological characteristics and were in a type of the donor plant from which they were obtained. Regenerants were doubled haploid plants, assessed visually by considering morphological characteristics and seed sets. The number of regenerants in each trial varied from 3 to 10. The GPRE rests on trial with the lowest value in A and the highest in H (Table 1).

**Table 1:** The arrangement of the induction medium composition and the time of anther culture contraposed with GPRE, metAFLP characteristics, and Fourier Transform Infrared (FTIR) data were used in the structural equation model for regenerants obtained in A-H trials.

Trial	*In vitro* anther culture conditions			metAFLP quantitative characteristics (%)		FTIR integrated absorbance (2,550–2,540 cm^−1^)	GPRE
	Cu (μM)	Ag (μM)	Time (days)	CHH_SV	CHH_DNMV	GSH	
A	0.1	10	42	8.66	0.37	0.004591	0.87
A	0.1	10	42	8.66	0.37	0.00494	0.87
A	0.1	10	42	8.52	0.36	0.005324	0.87
B	0.1	60	49	8.64	0.37	0.004879	1.52
B	0.1	60	49	8.64	0.37	0.00512	1.52
B	0.1	60	49	8.79	0.37	0.005144	1.52
B	0.1	60	49	8.79	0.37	0.004353	1.52
B	0.1	60	49	8.79	0.56	0.004985	1.52
C	5	60	42	8.76	0.75	0.004768	0.71
C	5	60	42	8.79	0.56	0.005038	0.71
C	5	60	42	8.64	0.55	0.00412	0.71
D	5	0	49	8.64	0.55	0.005176	2.38
D	5	0	49	8.64	0.55	0.005264	2.38
D	5	0	49	8.76	0.75	0.005502	2.38
D	5	0	49	8.76	0.75	0.00604	2.38
D	5	0	49	8.76	0.75	0.005451	2.38
D	5	0	49	8.76	0.75	0.005293	2.38
D	5	0	49	8.76	0.75	0.005133	2.38
D	5	0	49	8.76	0.75	0.00552	2.38
D	5	0	49	8.76	0.75	0.004793	2.38
D	5	0	49	8.91	0.76	0.005358	2.38
E	5	10	35	8.63	0.73	0.004881	1.17
E	5	10	35	8.63	0.73	0.004362	1.17
E	5	10	35	8.48	0.72	0.004652	1.17
E	5	10	35	8.48	0.72	0.005249	1.17
E	5	10	35	8.50	0.54	0.005302	1.17
F	10	10	49	8.48	0.54	0.005046	3.79
F	10	10	49	8.65	0.55	0.005121	3.79
F	10	10	49	8.65	0.55	0.005566	3.79
G	10	60	35	8.62	0.56	0.005394	4.24
G	10	60	35	8.49	0.55	0.005685	4.24
G	10	60	35	8.49	0.55	0.005823	4.24
G	10	60	35	8.65	0.55	0.005085	4.24
H	10	0	42	8.49	0.55	0.005692	6.06
H	10	0	42	8.49	0.55	0.005502	6.06
H	10	0	42	8.65	0.55	0.004594	6.06
H	10	0	42	8.65	0.55	0.005502	6.06

Fourteen-day-old leaves of the donor plant and its regenerants used for genomic DNA extraction resulted in DNA quality and quantity sufficient for the metAFLP analysis. Quantitative metAFLP analysis of banding patterns shared between donor plant and its regenerants showed SV within CHH asymmetric sequence context ranging from 8.48 to 8.91%, with the mean value equal to 8.65%. DNMV CHH context varied from 0.36 to 0.76% (mean value 0.58%). Similar characteristics for the CHG contexts related to DNMV, DMV, and SV ranged from 1.62 to 2.02% (CHG_DNMV), 2.86 to 2.94% (CHG_DMV), and 23.24 to 24.04% (CHG_SV). For the CG context, they were 0.87–1.34% (CG_DNMV), 1.33–1.50% (CG_DMV), and 11.35–11.96% (CG_SV). Only data concerning the CHH context implemented in the further analysis are arranged in Table 1.

**Figure 2:**
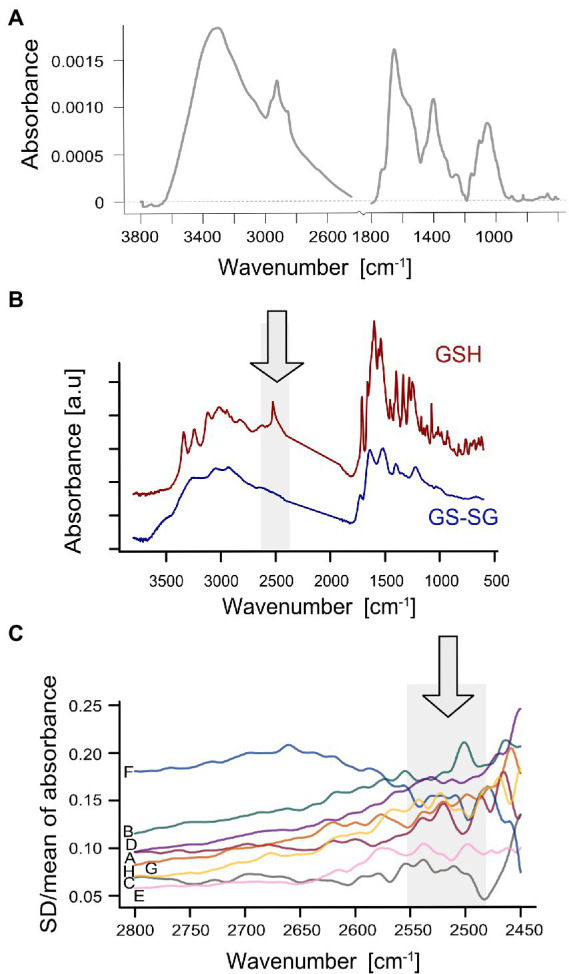
**(A)** Mean infrared spectrum of leaf tissues from the regenerants derived *via* anther cultures using eight experimental trials. **(B)** FTIR spectrum of glutathione in reduced (red) and oxidized form (blue). **(C)** The SD to mean ratio of absorbance for particular trails (labeled with letters).

The FTIR spectra of examined triticale leaf samples showed typical plant leaves pattern with characteristic massive broadband centered at 3,324 cm^−1^ corresponding to the C–H and the N–H bonds, the Amide I (*ca.* 1,645 cm^−1^), the complex band at 1,399 cm^−1^, and the carbohydrate fingerprint region between 1,200 and 900 cm^−1^ with maximum value at 1,048 cm^−1^ (Figure 2A). However, the spectral absorbance range between 1,800 and 2,400 cm^−1^ of absorbance was neglected from consideration due to the extensive interference of environmental dioxide and water vapor. To perform further analysis the absorbance values were summarized within successive 10 cm^−1^ intervals between 600–1,780 and 2,450–3,700 cm^−1^.

The infrared spectral characteristics of the glutathione reference sample in a solid state are given in Figure 2B. They show that the mid-infrared absorbance depended considerably on the oxidation states of glutathione. Major bands in GSH spectra corresponded to the stretching vibrations of the N–H (3,337, 3,240 cm^−1^), O–H (3,121 cm^−1^), C–H (2,947 cm^−1^), and S–H (c. 2,530 cm^−1^) bonds, the symmetric O–H stretching of COOH (2,907 cm^−1^), the C=O stretch in COOH (1,711 cm^−1^), and the symmetric stretching of COO– (1,395 cm^−1^). Similarly pronounced are the Amide I (1,660 cm^−1^), Amide II (*ca.* 1,538 cm^−1^), and Amide III (1,280 and 1,249 cm^−1^) bands (Picquart et al., 1999; Williams et al., 2009a; Tamasi et al., 2018). Most bands either disappeared, underwent reduction, or shifted in frequency for the oxidized form of glutathione.

The band around 2,550 cm^−1^ is not visible in the mean spectrum of leaf tissues, probably overlapped by commonly occurring broadband due to H-bonded O-H stretching vibrations from carboxylic groups of other compounds. To get more detailed graphical insight into this spectral region, we calculated SD/mean ratios for each experimental trial to evaluate possible spectral features variation for the wavenumbers, which could be attributed to GSH. Figure 2C shows the variability of this parameter within the spectral region between 2,450 and 2,800 cm^−1^. The spectral range at lower frequencies was discarded as disturbed by the presence of atmospheric dioxide.

Varying the metAFLP characteristics, including CHH_DNMV, CHG_DNMV, CG_DNMV, CHH_DMV, CHG_DMV, CG_DMV, CHH_SV, CHG_SV, and CG_SV were tested in the structural equation model. The same is valid for the FTIR spectral range from 2,600 to 2,450 cm^−1^, which was chosen based on the GSH standard FTIR spectrum. However, only those that resulted in significant output are presented.

The structural equation modeling analysis characteristics based on 37 samples shared between eight experimental trials are in Table 2. Skewness and kurtosis values indicate putative deviation from the normal distribution. Still, the variables were quantitative and met the conditions set out and the Lindeberg–Lévy theorem (Taboga, 2012). Thus, the distribution of these variables is assumed asymptotically convergent with the theoretical normal distribution.

**Table 2:** Descriptive statistics of the variables present in the postulated models.

Variable	Descriptive statistics			
	Mean	Variance	Skewness	Kurtosis
[Cu(II)]	5.43	12.785	−0.102	−1.008
[CHH_SV]	8.655	0.013	−0.079	−0.754
[CHH_DNMV]	0.584	0.019	−0.194	−1.054
Thiols [GSH]	0.005	0.000	−0.351	−0.054
[GPRE]	2.556	2.752	0.932	−0.122

Pearson’s linear correlation coefficients (Table 3) show that Cu(II) was negatively correlated with CHH_SV and positively with GSH and GPRE; GSH was positively correlated with GPRE. The other correlations were insignificant.

**Table 3:** Pearson’s linear correlation coefficients for analyzed variables.

Variable	[Cu(II)]	[CHH_SV]	[CHH_DNMV]	Thiols [GSH]	[GPRE]
[Cu(II)]	1				
[CHH_SV]	−0.386^*^	1			
[CHH_DNMV]	−0.32	0.247	1		
Thiols [GSH]	0.385^*^	−0.132	0.171	1	
[GPRE]	0.807^**^	−0.297	0.005	0.118^*^	1

* *p* ≤ 0.05;

** *p* ≤ 0.01.

**Figure 3:**
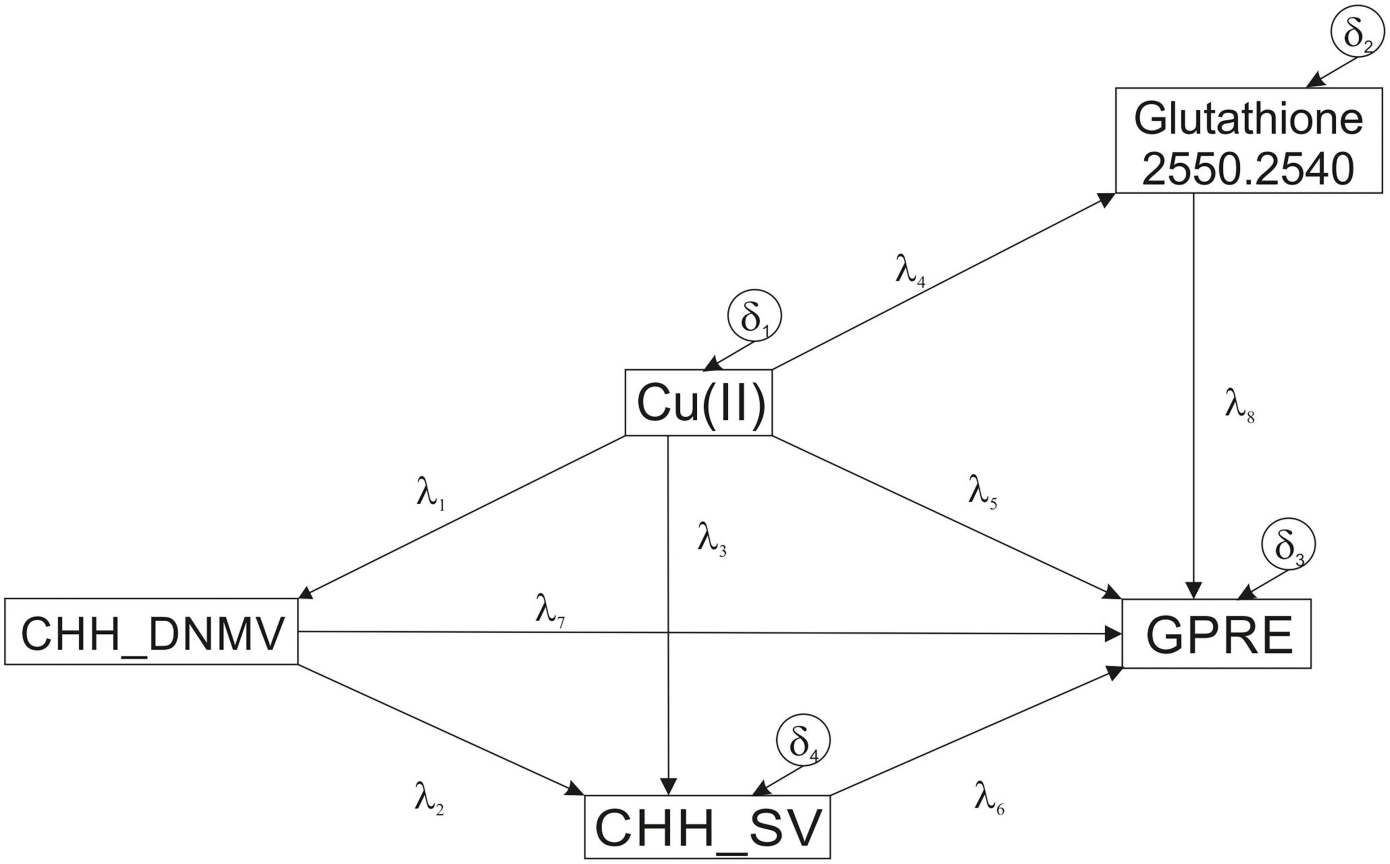
The hypothesized SEM model. Cu(II) ions concentration (μM); GPRE, green plant regeneration efficiency (number of regenerants per 100 plated anthers); the metAFLP quantitative characteristics concerning sequence variation (CHH_SV) and *de novo* DNA methylation (CHH_DNMV) between donor plant and its regenerants affecting asymmetric CHH sequence contexts; the 2,550–2,540 cm^−1^ is the FTIR spectrum wavenumber range (thiols give the univocal signal) indicating integrated absorbance used in the model; *λ*_1_–*λ*_8_ path coefficients, *δ*_1_–*δ*_4_ residuals (experimental errors).

The hypothesized model has a single exogenous variable (CHH_DNMV). The other variables were endogenous. All relationships were non-recursive. There were no covariances in the model. All variables were observed. The model included four residuals (Figure 3).

The Chi-square statistics (Table 4) usually used for the verification of fitting of the model is not significant. However, a restricted sample size (*n* = 37) used for the model construction may affect the outcome in the acceptance of an incorrect model (Kenny and McCoach, 2003). Therefore, following Mac Callum et al. (1996), the Chi-square model fit test was used as an information criterion. Thus, the model’s descriptive goodness-of-fit measures were used for the model fit evaluation. All the goodness-of-fit measures were within suggested ranges and indicated perfect fitting of the hypothesized model (Table 4). All paths’ (*b*) coefficients of the hypothesized model except the one for the CHH_SV → GPRE (Table 5) were significant.

**Table 4:** Summary of the analyzed structural equation model.

Parameter	Postulated model	Indices range	Recommended value for best fitting	References
Degrees of freedom (*df*)	2			
Chi-square (*χ*^2^)	0.11		Should be insignificant	
Value of *p*	0.95	0–1		
Root mean square residuals (RMR)	0.004		Closer to 0 is better	Joreskog and Sorbom, 1989; Jöreskog and Sörbom, 1993
Standardized root mean square residuals (SRMR)	0.014	0–1	≤ 0.08	Hu and Bentler, 1998, 1999; Wu et al., 2017
Goodness-of-fit index (GFI)	0.999	0–1	Closer to 1 is better	Mulaik et al., 1989
			≥ 0.95	Shevlin and Miles, 1998
			≥ 0.90	Wu et al., 2017
Adjusted goodness-of-fit index (AGFI)	0.991	0–1	Closer to 1 is better	Mulaik et al., 1989
			≥ 0.90	Hooper et al., 2008; Wu et al., 2017
Normed fit index (NFI)	0.999	0–1	≥ 0.90	Bentler and Bonett, 1980,
				Wu et al., 2017
			≥ 0.95	Hu and Bentler, 1998, 1999
Relative fit index (RFI)	0.993	0–1	≥ 0.95	Hu and Bentler, 1998, 1999
			≥ 0.90	Wu et al., 2017
Incremental fit index (IFI)	1.025		≥ 0.95	Hu and Bentler, 1998, 1999
			≥ 0.90	Hooper et al., 2008; Wu et al., 2017
Comparative fit index (CFI)	1	0–1	≥ 0.95	Hu and Bentler, 1998, 1999
			≥ 0.90	Hu and Bentler, 1998, 1999,
				Wu et al., 2017
Parsimonious normed fit index (PNFI)	0.2		Lower values indicate a greater models complexity	Hooper et al., 2008
Parsimonious comparative fit index (PCFI)	0.2		Lower values indicate a greater models complexity	Hooper et al., 2008
Root mean square error of approximation (RMSEA)	< 1 ∙ 10^−12^		≤ 0.05	Cudeck and Browne, 1992,
				Jöreskog and Sörbom, 1993
			< 0.05–very good fit	Mac Callum et al., 1996
			0.05–0.08–good fit	
			0.08–0.10–mediocre fit	
			> 0.10–poor fit	
			≤ 0.06	Hu and Bentler, 1999
			≤ 0.07	Steiger, 2007
			≤ 0.08	Wu et al., 2017

**Table 5:** Path coefficients, variances, and covariances for the analyzed model.

Parameter	Effect			Estimate (*b*)	Standard error	Test statistic	Standardized estimate (*β*)
*Path coefficients*							
*λ*_1_	[Cu(II)]	→	[CHH_DNMV]	0.0123	0.006	2.0256^*^	0.3199
*λ*_2_	[CHH_DNMV]	→	[CHH_SV]	0.3457	0.1230	2.8115^**^	0.4131
*λ*_3_	[Cu(II)]	→	[CHH_SV]	−0.0166	0.0047	−3.5252^***^	−0.5180
*λ*_4_	[Cu(II)]	→	[U2550.2540]	0	0	−2.5041^*^	0.3852
*λ*_5_	[Cu(II)]	→	[GPRE]	0.4249	0.0479	8.9650^***^	0.9132
*λ*_6_	[CHH_SV]	→	[GPRE]	2.4888	1.3685	1.8187	0.1715
*λ*_7_	[CHH_DNMV]	→	[GPRE]	−4.4056	1.1149	−3.9515^***^	−0.3628
*λ*_8_	[U2550.2540]	→	[GPRE]	752.6512	334.1213	2.2526^*^	0.1923
*Variances*							
*δ*_1_				0.0164	0.60039	4.24326^***^	
*δ*_2_				0.6014	0.1417	4.2426^***^	
*δ*_3_				0.0089	0.0021	4.2426^***^	
*δ*_4_				0	0	4.2426^***^	

* *p* ≤ 0.05;

** *p* ≤ 0.01;

*** *p* ≤ 0.001.

The most critical path described the relationships between Cu(II) and GPRE, followed by the one for Cu and CHH_SV as indicated by standardized path coefficients (*β*) values of the hypothesized model. In addition, the relationships between CHH_DNMV and CHH_SV and between Cu(II) and GSH (U2550.2540) were also significant with high coefficient values.

The model can be decomposed into direct, indirect, and total effects (Table 6). The GPRE variable showed the most significant dependence on Cu(II) (total *β* = 0.8051; including the direct effect as *β* = 0.913, and the indirect effect as *β* = −0.1082). CHH_SV depended to the greatest extent on Cu(II) (direct *β* = −0.518, indirect *β* = 0.1321, and total *β* = −0.3859 effects). On the other hand, GSH (U2550.2540) was affected by Cu (II) (direct and total *β* = 0.3852 effects) whereas Cu(II) by CHH_SV (*β* = 0.32 for direct and total effects).

**Table 6:** Direct, indirect, and total effects for the analyzed model.

Effect			Estimates (*b*)			Standardized Estimates (*β*)		
			Direct effect	Indirect effect	Total effect	Direct effect	Indirect effect	Total effect
[GPRE]								
[Cu(II)]	→	[GPRE]	0.4249	−0.0503	0.43745	0.9132	−0.1082	0.8051
[CHH_DNMV]	→	[GPRE]	−4.4056	0.8604	−3.5452	−0.3628	0.0708	−0.2919
[U2550.2540]	→	[GPRE]	752.6512	0	752.6512	0.1923	0	0.1923
[CHH_SV]	→	[GPRE]	2.4888	0	2.4888	0.1715	0	0.1715
[CHH_SV]								
[Cu(II)]	→	[CHH_SV]	−0.0166	0.0062	−0.0124	−0.518	0.1321	−0.3859
[CHH_DNMV]	→	[CHH_SV]	0.3457	0	0.3457	0.4131	0	0.4131
[U2550.2540]	→	[CHH_SV]	0	0	0	0	0	0
[U2550.2540]								
[Cu(II)]	→	[U2550.2540]	0	0	0	0.3852	0	0.3852
[CHH_DNMV]	→	[U2550.2540]	0	0	0	0	0	0
[CHH_DNMV]								
[Cu(II)]	→	[CHH_DNMV]	0.0123	0	0.0123	0.3199	0	0.3199

## Discussion

In the experiment presented here, the metAFLP technique was used along with specially dedicated plant material to estimate tissue culture-induced variation. Although obtaining regenerants was conducted from 24 donor plants, identical in terms of morphological traits, only regenerants from a single donor plant that allowed regeneration of doubled haploid plants through all trials were finally selected for the study. Such an approach (Pachota et al., 2022) was selected to avoid even minor (epi)genetic (pre-existing) variation that persisted between donors and might have resulted in genotype (donor) effect when obtaining plants *via in vitro* cultures (Flinn et al., 2020). The abundance of regenerants for each trial (A-H) varied (3–10), which is due to the very specificity of plants’ obtention *via in vitro* systems and the difficulties that the process of androgenesis carried out in anther cultures from microspores is burdened with (e.g., low frequency of spontaneous genome doubling).

Despite the lack of morphological differences between donor plants and their regenerants derived *via* several experimental conditions encompassing different concentrations of Cu(II), Ag(I), and time of anther culture, the metAFLP approach revealed the presence of differences in the DNA sequence and DNA methylation levels. In the case of CHH contexts, DNMV increased in regenerants compared to the donor plant and somewhat depended on the anther culture conditions. DNMV was the lowest when Cu(II) in the IM was the lowest and the highest when Cu(II) was in its middle concentration. The time of anther culture and Ag(I) seem not to influence *de novo* methylation. Sequence variation affecting CHH context was relatively high independently of the experimental conditions with the highest values for trial “D” (see Table 1). The presented observations are congruent with our earlier studies on triticale anther cultures (Orłowska et al., 2022) where DNMV rather than DMV were evaluated. We tend to think that such a change reflects cell dedifferentiation (Fehér, 2019) and the cell fate reprogramming (Fehér, 2015; Elhiti et al., 2018; Jing et al., 2020) from gametophytic to sporophytic fate (Testillano, 2019).

Among several absorbance bands in the FTIR spectra, the one located around 2,550 cm^−1^ may be considered the most specific for GSH since it occurs out of the fingerprint region. This band is a distinguishing feature of glutathione in reduced form (GSH) and may be assigned to S–H stretching vibrations (Picquart et al., 1999; Williams et al., 2009a; Tamasi et al., 2018). Thus, the spectral range 2,550–2,540 cm^−1^ encompassing the local maximum on the reference was used for future purposes. Taking into account the results from FTIR data for GSH/GSSG (Figure 2B) and data available in the literature (Picquart et al., 1999; Tamasi et al., 2018), it was assumed that the region is characteristic for thiols. Furthermore, assuming that the most representative thiol in the cell (García-Giménez et al., 2017) is GSH, the FTIR range was assigned to the compound. For sure, it would be of value to conduct either enzymatic (Monostori et al., 2009) or mass spectroscopy (MS) analysis (Iglesias González et al., 2015) to have independent confirmation that the FTIR spectra reflect GSH. Unfortunately, we could not perform such an analysis due to insufficient plant materials. Still, we believe that using FTIR spectra for GSH standard indicated regions that should be considered indicative of the compound.

We did not observe this peak in the mean spectrum of leaves, as vibrations from compounds with H-bonded carboxylic groups and overtones probably overlapped (Giubertoni et al., 2020). However, the infrared analysis of plant tissues collected from different trials showed a significant variability of the spectral features evaluated for all experimental trials in this region, particularly between 2,550 and 2,500 cm^−1^, when the SD of the absorbance normalized to the mean was taken into account. This graphically illustrated variability may indicate that the reduced to oxidized glutathione ratio varied across the trials.

GSH may form complexes with copper (complex Cu(I)-glutathion) (Speisky et al., 2008). Thus, copper ions in the IM may affect the glutathione-ascorbate cycle and, consequently, efficient ROS elimination. Interestingly, under the IM conditions resulting in the highest mean values of sequence variation and DNA *de novo* methylation [middle Cu(II) concentration in the IM and the longest time of anther cultures], the FTIR peak values seem to be the highest, suggesting an increased role of thiols (and specifically GSH) under such experimental conditions.

Glutathione may function as a second messenger in cells and a modifier of the histones. Furthermore, GSH may control epigenetic mechanisms at substrate availability, DNA methylation, and expression of microRNAs. However, the molecular pathways by which GSH controls epigenetic events are not evident (García-Giménez et al., 2017). It is also the truth in the case of anther culture regarding tissue culture-induced variation and the efficiency of green plant regeneration. As epigenetic mechanisms reestablish CHH sequence context DNA methylation, those contexts were the primary targets of our analysis to evaluate relationships between TCIV characteristics, IM composition, and GPRE.

A structural equation model was implemented to evaluate relationships between numerous variables influencing GPRE in triticale. All analyzed variables met the Lindeberg–Lévy theorem (Taboga, 2012). Therefore, it can be assumed that the distribution of these random variables is asymptotically convergent with the normal distribution. The fulfillment of this assumption means that the covariance matrix from the analyzed sample has the Wishart distribution. The distribution is considered a detailed statistic and is the optimal estimator of the covariance matrix in the general population in the SEM analysis (Mooijaart and Bentler, 1986; Johnson and Wichern, 1992). Observed increased skewness of some variables, such as GRPE (Table 2), may contribute to an increase in the value of the Chi-square statistic used in the SEM analysis to assess the significance of the postulated model and may contribute to an increase in measures of absolute model fit (Bentler and Bonett, 1980; Kenny, 2019).

Additionally, small sample size may result in a falsely irrelevant Chi-square statistic (Kenny and McCoach, 2003). It should also be noted that since the data come from a natural experiment, where the assumption that the randomness effect is quite significant, the problem of determining the significance of Chi-square statistics often arises (Vargas et al., 2007) is due to the tendency of the Chi-square test to give a false-significant test result when the postulated model is not an ideal representation of all relationships occurring in the general population (Mac Callum et al., 1996). Therefore, it is recommended (Mac Callum, 2003) that the Chi-square statistic instead be used as an information criterion on an equal footing with other measures of fit in the postulated model.

The covariance matrix is the primary source of information about the system of mutual relations in the empirical data set in the SEM analysis. Its standardized form is the correlation matrix. In the analyzed case, the values of the Pearson’s linear correlation coefficients included in this matrix (Table 3) prove the interrelationships between the analyzed variables. However, these relationships are of different strengths and nature. Thus, single regression models or Wright’s path analysis (Wright, 1921, 1923, 1934) is not an appropriate tool to describe a rather complex system of relations between the analyzed variables. A reasonable solution, in this case, is to try to build a model based on the SEM methodology.

Apart from the above, in assessing the fit of the postulated model, attention should be paid to the possible effect of sample size on the estimated fit measures. Sivo et al. (2006) showed that with the decrease in the sample size, the value of some measures of fit decreased (GFI, AGFI, CFI, NNFI, NFI, PGFI, and PNFI), and the value of some measures increased (RMR, SRMR, RMSEA). However, the values of the measures fitting the postulated model reached the limit thresholds (Table 4), indicating a perfect fit of the model to the empirical data set. The notion is confirmed by the absolute (GFI, AGFI, RMR, and SRMR) and the relative fit measures (NFI, RFI, IFI, and CFI). Also noteworthy is the shallow value of the model approximation error (RMSEA), reflecting a perfect model fitting. However, small values of PNFI and PCFI indices (reflect the complexity of the model) indicate that the model is a bit complex and possibly should be simplified unless removing individual relations or variables from the model deteriorates the remaining adjustment measures.

Based on the analysis of the postulated model (Table 5), it could be concluded that all relations described by the model (model paths), except the direct relationship between CHH_SV and GPRE, were statistically significant. However, the elimination of the path resulted in a decrease in fit indices. Assuming that the path was demonstrated significant in other similar analyses, it was not deleted from the model. The most important relationship was the dependence of GRPE on Cu(II) and CHH_SV on Cu(II), which most probably reflects copper’s role in acting as a cofactor of many enzymatic reactions in biochemical pathways. The other significant path (CHH_DNMV → CHH_SV) supports the notion that DNA demethylation (Gong and Zhu, 2011; Li et al., 2018) may follow an alternative pathway similar to that in mammals. The 5mC oxidation process (Kreutzer and Essigmann, 1998; Tang et al., 2014) or deamination can alter coding sequences (Nabel et al., 2012) and result in sequence variation. The interpretation is even more vital as the asymmetric sequence context encompasses the model, while other contexts failed to build the model. We think that the result reflects the epigenetic aspects of CHH methylation pattern reestablishment (He et al., 2011; Matzke and Mosher, 2014; Zhang et al., 2018), which may increase mutations within the sequence type.

Decomposing the combined effects in the analyzed model into a direct and indirect effect (Table 6) allows for a detailed examination of the interdependencies of individual variables directly and by interacting through intermediary variables. Such an approach allows, for example, to identify a relationship for which direct and indirect effects cancel out (the dependence of GRPE on CHH_DNMV), making the total effect appear small and unimportant.

Possibly, the disadvantage of the study is a small sample size. However, the experimental model assumes that all regenerants should be derived from the same donor plant to avoid (epi)genetic variation of donors and to eliminate the pre-existing variation. To obtain a large sample size in all trials is a rather tricky task, even in the case of triticale anther culture. Despite the evident pros of the approach, its drawback was the number of regenerants that could be regenerated simultaneously in all trials. Thus, we expected to identify exclusively strong relationships, while minor ones will not be captured or insignificant. Furthermore, the model should have a biological background. Otherwise, it could not be treated as reflecting natural phenomena.

The presented model fits the biological understanding of TCIV. To our best knowledge, the study is the first indication of the multiple impacts of Cu(II) on biochemical cycle(s) and pathways that participate in (epi)genetic regulation of GPRE due to *in vitro* anther tissue cultures. The epigenetic aspect of GPRE is reflected by DNA *de novo* methylation within asymmetric CHH contexts involving copper ions. Furthermore, copper ions interact with GSH, most likely affecting the cell redox and impacting the epigenetic mechanisms leading to GPRE, which agrees with available data (García-Giménez et al., 2017). A significant role of the GSH in plant regeneration *via* anther cultures was also shown in triticale (Żur et al., 2019) and rye (Zieliński et al., 2020). Stressing tillers before plating explants (Żur et al., 2019; Zieliński et al., 2020) increased the final effectiveness in microspore embryogenesis of recalcitrant rye lines or increased the number of embryo-like structures in triticale, but the mechanism was not evaluated. While, the addition of GSH to IM caused changes in the level of oxidative stress level affect auxin production, which triggers embryogenic development in cultured Arabidopsis explants (Kudełko and Gaj, 2019). Based on the current study, it is hardly difficult to show directly what epigenetic mechanism are involved here. However, their significance in GPRE seems evident. Furthermore, GPRE seems to involve some genetic aspects as sequence variation also participated in GPRE. However, this feature was shallow. Possibly, the primary output from the current study is that varying Cu(II) concentration in the IM one may control for the GPRE and that Cu(II) may increase the number of green plants if the adequate concentration of Cu(II) in the IM is available. Furthermore, Cu(II) seems to be a crucial checkpoint of the GPRE linking DNA methylation, sequence variation, and GSH.

## Conclusion

A structural equation model reflecting complex relationships between sequence variation, *de novo* DNA methylation within asymmetric sequence contexts, and copper ions in the IM, as well as, GSH, and GPRE, was evaluated. Statistical analysis showed that the postulated model perfectly fits experimental data. Furthermore, it reflects biological phenomena in the cell. Still, we expect that the identified relationships reflect exclusively strong effects, while minor ones are omitted due to the relatively small sample size. An exciting aspect of the study is the indication of the input of GSH to GPRE. Considering its putative key role in epigenetics of the cell, we speculate that GSH in anther cultures, most probably *via* not fully recognized epigenetic processes, impacts green plant regeneration efficiency.

Furthermore, the central role of copper ions should be mentioned. Being a cofactor of many enzymatic reactions, they may influence biochemical pathways affecting tissue culture-induced variation, GSH, and GPRE. The model assumes that asymmetric sequence contexts (CHH) participate in GPRE, indicating the importance of epigenetics in anther tissue cultures of triticale. Despite scientific meaning, the presented model also has practical implications showing that manipulating copper ion concentration in the IM makes it possible to manipulate cell functioning and increase the GPRE.

## Data availability statement

The original contributions presented in the study are included in the article/Supplementary material, further inquiries can be directed to the corresponding author.

## Author contributions

PB: idea of the study, statistical analysis, model interpretation, and writing the manuscript. RO: idea of the study, run molecular analysis and participated in FTIR experiment, and participated in data analysis and manuscript writing. DM: described part of the data concerning SEM and participated in manuscript writing. KK and MN: double-checked metAFLP markers and converted them into a binary matrix. JZi: participated in plant material evaluation and paper writing. JZe: conducted FTIR data analysis, their interpretation, and participated in writing the manuscript. All authors contributed to the article and approved the submitted version.

## Funding

This research was funded by the Ministry of Agriculture and Rural Development, Poland (grant no. HORhn-801-PB-22/15–18).

## Conflict of interest

The authors declare that the research was conducted without any commercial or financial relationships construed as a potential conflict of interest.

## Publisher’s note

All claims expressed in this article are solely those of the authors and do not necessarily represent those of their affiliated organizations, or those of the publisher, the editors and the reviewers. Any product that may be evaluated in this article, or claim that may be made by its manufacturer, is not guaranteed or endorsed by the publisher.

## Supplementary material

The Supplementary Material for this article can be found online at: https://www.frontiersin.org/articles/10.3389/fpls.2022.926305/full#supplementary-material

## Abbreviations

ATR: Attenuated total reflectance
CMT3: chromomethylase 3 (CMT3)
Cu/Zn SOD: copper/zinc superoxide dismutase
DArTseqMet: Diversity arrays technology sequencing methylation analysis
DMV: demethylation
DNMV: *de novo* methylation
DTGS: deuterated triglycine sulfate
FTIR: Fourier transform infrared spectroscopy
GPRE: green plant regeneration efficiency
GSH: glutathione
metAFLP: methylation-sensitive amplified fragment length polymorphism
NGS: next-generation sequencing
RdDM: RNA-directed DNA methylation
RNS: reactive nitrogen species
ROS: reactive oxygen species
SAM: S-adenosyl-L-methionine
SEM: structural equation modeling
SV: sequence variation
TCIV: tissue culture-induced variation
