# Corrigendum: Glutathione and copper ions as critical factors of green plant regeneration efficiency of triticale *in vitro* anther culture

**DOI:** 10.3389/fpls.2022.1081635

**Published:** 2022-11-17

**Authors:** Piotr T. Bednarek, Renata Orłowska, Dariusz R. Mańkowski, Janusz Zimny, Krzysztof Kowalczyk, Michał Nowak, Jacek Zebrowski

**Affiliations:** ^1^ Plant Breeding and Acclimatization Institute–National Research Institute, Radzików, Poland; ^2^ Institute of Plant Genetics, Breeding and Biotechnology, University of Life Sciences in Lublin, Lublin, Poland; ^3^ Institute of Biology and Biotechnology, University of Rzeszow, Rzeszow, Poland

**Keywords:** androgenesis, copper, glutathione, regeneration efficiency, triticale

In the published article, the affiliations were incorrect. Dariusz R. Mańkowski’s affiliation was incorrectly published as affiliation 2 “Department of Applied Biology, Plant Breeding and Acclimatization Institute—National Research Institute, Radzików, Poland”. This affiliation has been removed and Dariusz R. Mańkowski’s affiliation is now affiliation 1. The correct affiliation details appear as follows:

Piotr T. Bednarek ^1†^, Renata Orłowska ^1*†^, Dariusz R. Mańkowski ^1^, Janusz Zimny ^1^, Krzysztof Kowalczyk ^2^, Michał Nowak ^2^ and Jacek Zebrowski ^3^

^1^ Plant Breeding and Acclimatization Institute–National Research Institute, Radzików, Poland,

^2^ Institute of Plant Genetics, Breeding and Biotechnology, University of Life Sciences in Lublin, Lublin, Poland,

^3^ Institute of Biology and Biotechnology, University of Rzeszow, Rzeszow, Poland.

The authors apologize for this error and state that this does not change the scientific conclusions of the article in any way. The original article has been updated.

## Publisher’s note

All claims expressed in this article are solely those of the authors and do not necessarily represent those of their affiliated organizations, or those of the publisher, the editors and the reviewers. Any product that may be evaluated in this article, or claim that may be made by its manufacturer, is not guaranteed or endorsed by the publisher.

