# Supplementary figures and images for: Glutathione and copper ions as critical factors of green plant regeneration efficiency of triticale in vitro anther culture
Source: Front Plant Sci. 2022 Jul 28;13:926305. doi: 10.3389/fpls.2022.926305 (PMC9379855; doi:10.3389/fpls.2022.926305)

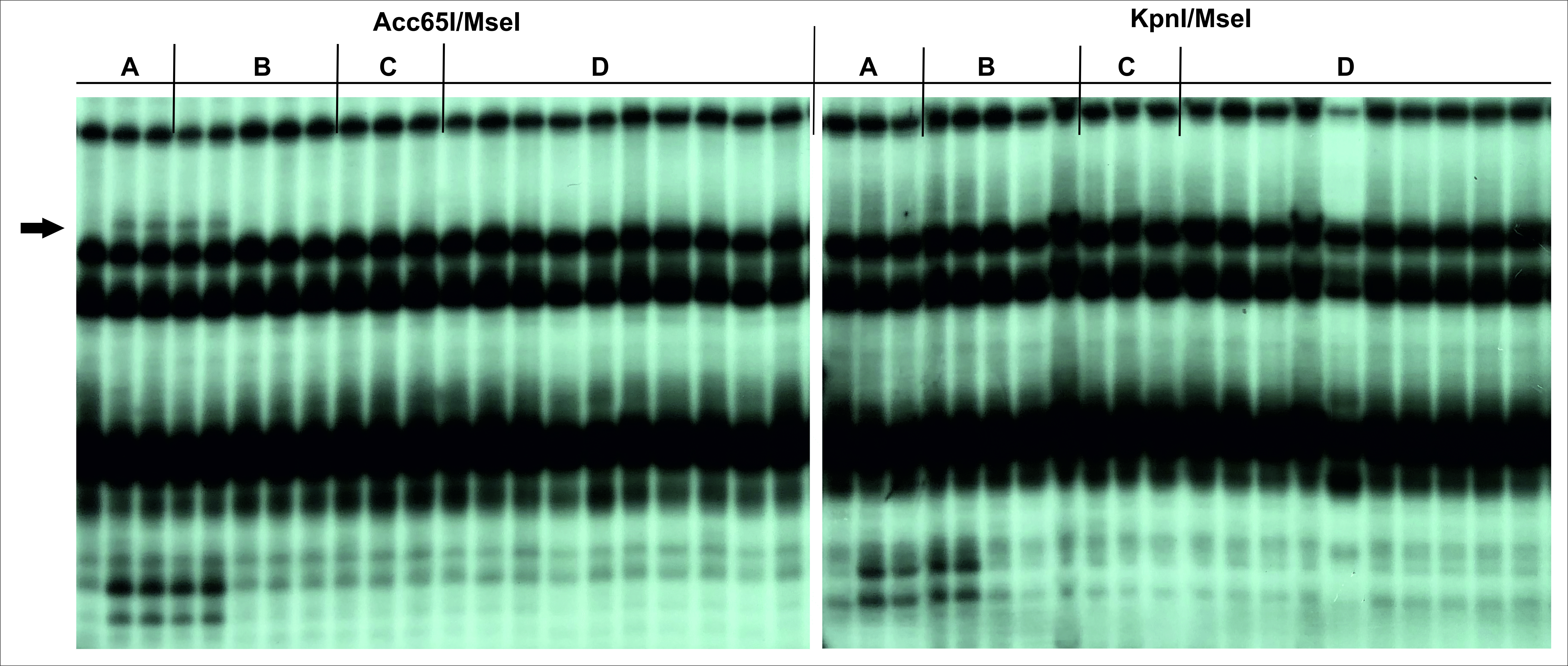

Supplement: Supplementary file 1 [file Image_1.JPEG]
